# The impact of telework intensity on employee proactive behavior: based on the uncertainty management theory

**DOI:** 10.3389/fpsyg.2026.1854454

**Published:** 2026-10-29

**Authors:** Yixin Zhang, Weipeng Lin, Mengli Liu, Huijuan Wang

**Affiliations:** 1 School of Management, Shandong University, Jinan, China; 2 Xuzhou College of Industrial Technology, Xuzhou, China; 3 Inspur Digital Enterprise Technology Limited Corporation, Jinan, China

**Keywords:** media richness, media richness theory, proactive behavior, telework intensity, uncertainty management theory

## Abstract

**Purpose:**

This study investigates how telework intensity differentially influences three theoretically differentiated foci of employees’ proactive behavior, drawing on uncertainty management theory and media richness theory. It specifically examines the mediating role of perceived uncertainty and explores how media richness of communication technologies serves as a critical boundary condition in this process.

**Design/methodology/approach:**

A two-wave, matched leader-employee survey was conducted, collecting data from 212 employees and 81 supervisors across multiple provinces in China. The proposed moderated mediation model was analyzed using SPSS and Mplus.

**Findings:**

Results reveal that telework intensity directly increases employees’ perception of uncertainty, which in turn indirectly reduces prosocial and pro-organization foci proactive behaviors, but does not significantly affect pro-self foci proactive behavior. Media richness significantly moderates this effect: higher media richness weakens the positive relationship between telework intensity and the perception of uncertainty.

**Originality:**

This study shifts the focus in telework research from general outcomes to a nuanced examination of proactive behavior across distinct foci. It introduces and empirically validates media richness as a pivotal technological moderator, clarifying both how telework intensity influences employees and under what technological conditions its effects are attenuated.

## 1 Introduction

The COVID-19 pandemic accelerated the shift to telework, in which employees work outside traditional offices using telecommunications technology. Telework is characterized by geographical dispersion and technology-dependent communication, offering employees temporal and locational flexibility (Golden and Veiga, 2005; Kumar et al., 2025). These advantages have transformed telework from a temporary solution into a permanent feature of modern work, driven by sustained employee preferences and increased inter-organizational collaboration (Boyraz and Gilbert, 2024).

The growing prevalence of telework has attracted considerable scholarly attention. Prior work has largely drawn on relational or job characteristics perspectives to examine its effects on relatively distal outcomes, such as work attitudes (Gajendran and Harrison, 2007; Golden and Veiga, 2005), communication and relationship quality (Lal et al., 2023; Wöhrmann and Ebner, 2021), performance (Leroy et al., 2021; Golden and Eddleston, 2020), work-life balance (Bellmann and Hübler, 2021; Palumbo, 2020; Zürcher et al., 2021), behavior and well-being (Ferdous et al., 2026; Nowrouzi-Kia et al., 2024). Other studies have focused on more proximal psychological states, including isolation and loneliness (Taser et al., 2022; Van Zoonen and Sivunen, 2022), role ambiguity and conflict (Sardeshmukh et al., 2012), pressure from time or technology (Blind et al., 2024; Darouei and Pluut, 2021; Taser et al., 2022; Wöhrmann and Ebner, 2021), perceptions of disturbances and interruptions (Leroy et al., 2021; Wöhrmann and Ebner, 2021), autonomy (Gajendran et al., 2015; Müller and Niessen, 2019; Vander Elst et al., 2017) basic psychological needs satisfaction (Brunelle and Fortin, 2021), social support perception (Huo et al., 2023; Kröll et al., 2021; Vander Elst et al., 2017), and work exhaustion (Golden, 2006a). These variables capture single and specific psychological states, each targeting a concrete domain such as relationships, roles, or resources.

While offering valuable insights, these research overlooks work environment uncertainty as a theoretical mechanism. This omission limits understanding because distance is a defining feature of telework (Leonardi et al., 2024). Telework presents a core paradox: the same flexibility that empowers employees also creates psychological distance (cognitive, affective, or social separation; e.g. lack of social connectedness), temporal distance (separation in time; e.g., delayed feedback), and structural distances (misalignments in organizational or administrative demands; e.g. blurred work-nonwork boundaries; Leonardi et al., 2024), fostering the perception of uncertainty (PU), which stems from insufficient external information or unclear self-perceptions (Van den Bos, 2001). Importantly, PU represents a general psychological state regarding the overall work environment, and thus can more fully capture how telework, serving as a mode of work characterized by multiple forms of distance, affects employees’ psychological mechanisms.

PU is particularly consequential for employee proactive behavior, which is future-oriented action that is inherently riskier than routine behaviors and thus more susceptible to uncertainty. However, existing research has treated proactive behavior as a monolithic construct, focusing on specific outcomes such as innovation (Huo et al., 2023) or knowledge sharing (Peng and Chen, 2022) and failing to account for how telework-induced PU may differentially impact behaviors directed toward the organization (pro-organizational foci proactive behavior, POPB), toward colleagues (prosocial foci proactive behavior, PSPB), and toward oneself (pro-self foci proactive behavior, PFPB). These distinctions matter because each focus entails different levels of interpersonal risk and external visibility. To address this gap, we draw on uncertainty management theory to investigate how telework intensity influences each proactive behavior. Accordingly, the aim of this study is to examine the differential effects of telework intensity on employees’ proactive behaviors from the perspective of uncertainty management theory, including firstly examining the effect of telework intensity on PU, and subsequently how telework intensity influences the three foci proactive behaviors with PU serving as a mediator.

Additionally, recognizing the critical role of communication technology in telework, we further examine media richness, referring to the capacity of a communication medium to convey information (Daft and Lengel, 1984, 1986), as a moderator. Telework’s heavy reliance on technology makes media richness an inevitable contextual factor (Shockley et al., 2021). Prior research has focused on work-family boundary conditions (e.g., autonomy, family size, Golden et al., 2006), while technology-related factors have received relatively less attention in the specific context of telework intensity and perceived uncertainty. Although media richness has been examined in relation to virtual teams and distance working more broadly (Cheung et al., 2024; Kaplan et al., 2018), its role as a boundary condition in the telework-uncertainty linkage remains underexplored. Thus, drawing on media richness theory (Daft and Lengel, 1984, 1986), we propose that media richness attenuates the effect of telework. Accordingly, another aim of this study is to establish the moderating role of media richness on the relationship between telework intensity and PU, as well as subsequent different-foci proactive behavior.

Our study offers several contributions. Theoretically, we introduce PU as a novel mediating mechanism linking telework intensity to proactive behavior, extending telework research into the uncertainty domain. We further consider three foci of proactive behavior, including organization-directed, colleague-directed, and self-directed, rather than treating proactive behavior as a monolithic construct, and demonstrate that telework-induced uncertainty primarily impairs these behaviors. We also identify media richness as a boundary condition, addressing the overlooked role of technological context. Practically, our findings underscore the importance of designing telework policies that mitigate psychological uncertainty and enhance the media richness of communication channels. In summary, our theoretical framework is grounded in uncertainty management theory and media richness theory, and integrates telework intensity, PU, three different foci proactive behaviors and media richness. The proposed model is presented in Figure 1.

**Figure 1:**
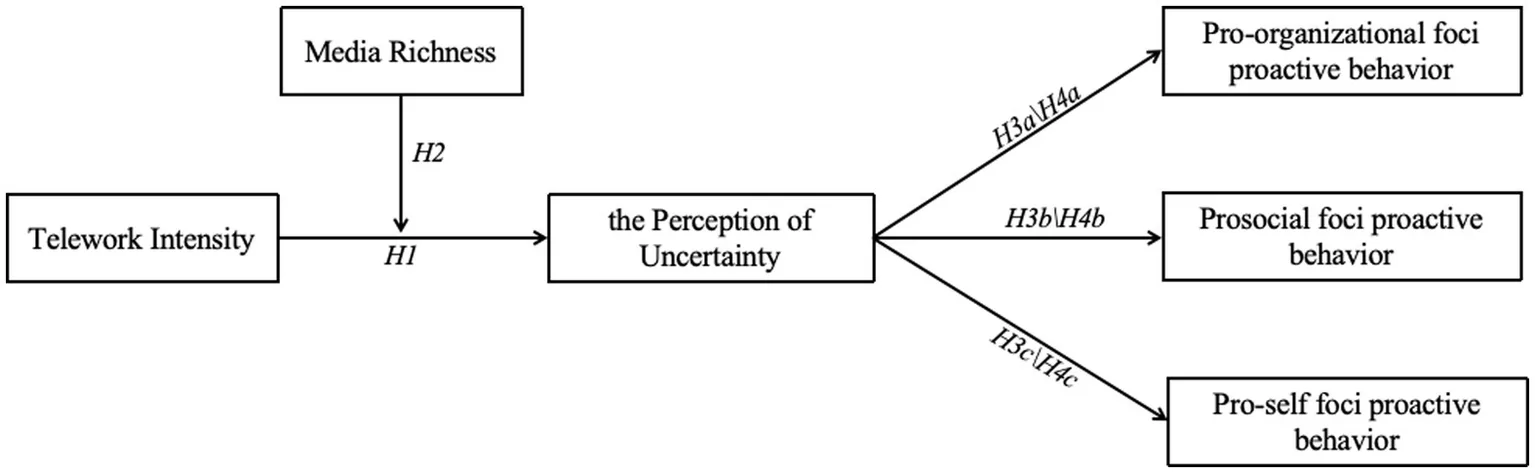
Theoretical model.

## 2 Theory and hypotheses

Grounded in uncertainty management theory, we develop our hypotheses by following its core theoretical logic. Following the theoretical rationale, we first address how telework intensity triggers perceived uncertainty and how media richness moderates this triggering process. Building on this, we then examine how perceived uncertainty differentially drives three foci proactive behavior. Finally, we integrate the above stages into a complete moderated mediation model.

### 2.1 The effect of telework intensity on PU

According to uncertainty management theory, PU originates from two sources: insufficient information for forming social judgments and internal doubts about self-perception and worldview (Van den Bos and Lind, 2013). Telework’s geographic dispersion and technological reliance (Golden and Veiga, 2005) may trigger both sources of uncertainty by altering the work environment and limiting information flow (Taskin and Bridoux, 2010). Thus, uncertainty management theory provides a theoretical framework for understanding employees’ feelings and reactions to telework intensity.

First, telework intensity reduces direct physical contact with the organization, limiting employees’ access to organizational information compared to office-based colleagues. Teleworkers rely heavily on electronic media to communicate, which constrains their ability to obtain nonverbal cues such as body language and facial expressions (Golden, 2006b). Thus, the richness and quality of information for decision-making is reduced, causing employees to experience uncertainty (Taskin and Bridoux, 2010).

Second, the physical isolation inherent in telework makes it difficult for leaders and coworkers to observe day-to-day performance, potentially leading to underestimation of employee commitment and effort (Golden and Eddleston, 2020). This lack of visibility may cause employees to feel “invisible to the company” and doubt whether their efforts will be accurately evaluated for rewards and promotion (Taskin and Bridoux, 2010). Such doubts prompt employees to question their self-perception regarding work effort, which according to uncertainty management theory constitutes another source of uncertainty (Van den Bos, 2001). Thus, we hypothesize:

*H1:* Telework intensity has a positive effect on PU.

### 2.2 The moderation effect of media richness

Media richness refers to a communication medium’s capacity to convey information (Daft and Lengel, 1984, 1986) and represents a key situational factor in technology-mediated telework. Media richness theory posits that communication aims to reduce uncertainty and ambiguity, and that rich media achieve this by enhancing information quality and transmission efficiency (Daft and Lengel, 1984, 1986). Drawing on this theory, we propose that media richness moderates the relationship between telework intensity and PU.

High media richness is characterized by immediate feedback, multiple cues, language variety, and personal focus (Ishii et al., 2019). These features enhance information quality and richness in telework communication (Kaplan et al., 2018). Specifically, multiple cues such as vocal inflection, gestures, and facial expressions enable employees to accurately interpret others’ emotions and intentions, facilitating deeper understanding (Dennis and Kinney, 1998). Language variety allows for conveying complex concepts and ideas (Braun et al., 2019), while personal focus aids emotional communication (Dennis and Kinney, 1998). Additionally, immediate feedback provides quick access to others’ decisions, helping employees understand work directions (Chidambaram and Jones, 1993), and reducing concerns that their efforts go unnoticed. Thus, high media richness mitigates the communication challenges arising from geographic dispersion and technological dependence in telework by improving both the quality and efficacy of information exchange.

Consequently, high media richness weakens the positive relationship between telework intensity and PU. Conversely, low-richness media reduce communication effectiveness and message comprehension (Daft and Lengel, 1984, 1986), strengthening this relationship. Thus, we hypothesize:

*H2:* Media richness moderates the relationship between telework intensity and PU such that the positive relationship is weaker when media richness is high rather than low.

### 2.3 The effect of PU on proactive behavior

Uncertainty management theory posits that PU motivates individuals to reduce it, often through avoidance strategies, and may also prompt individuals to take proactive steps to restore predictability (Hogg, 2007; Van den Bos, 2001). Accordingly, based on the distinct characteristics of different foci of proactive behavior, as well as their respective targets and associated risks, we propose that perceived uncertainty differentially affects three theoretically differentiated foci of proactive behavior.

POPB refers to self-starting, future-oriented actions aimed at benefiting the organization (Belschak and Den Hartog, 2010). Such behavior entails considerable personal risk, as failure may result in negative performance evaluations, criticism for deviating from instructions, or the perception of low commitment (Belschak and Den Hartog, 2010). According to uncertainty management theory, employees perceiving uncertainty will reduce POPB to avoid these potential negative outcomes and the further uncertainty they would generate (Lind and Van den Bos, 2002).

Similarly, PSPB is directed toward teams and colleagues, encompassing knowledge sharing, skill assistance, and supportive actions (Belschak and Den Hartog, 2010; Belschak et al., 2010). When employees experience uncertainty, they lack clear understanding of team members’ expectations and social norms. This ambiguity may lead them to worry that prosocial initiatives could be misinterpreted or perceived negatively, or even that they might be misinterpreted as arrogance (Parker et al., 2010; Parker and Collins, 2010). Consequently, uncertainty management theory indicates that employees withdraw from PSPB to avoid interpersonal situations that might exacerbate uncertainty (Van den Bos and Lind, 2013).

In contrast, PFPB centers on personal development, involving active efforts to acquire new knowledge, improve work methods, and pursue career goals (Belschak and Den Hartog, 2010). Unlike POPB and PSPB, which rely on external feedback and organizational recognition that are inherently unpredictable, PFPB targets the self and its outcomes are largely within the employee’s own control. When employees perceive uncertainty, uncertainty management theory suggests that such perceptions diminish their sense of control, motivating them to restore predictability (Hogg, 2007). One effective way to regain predictability is to focus on domains that are directly manageable. Specifically, by proactively seeking work-related information, acquiring new skills, and improving personal work methods, employees can enhance their personal resources and competitiveness (Yang et al., 2023; Guo, 2011). In doing so, these tangible personal achievements restore career-related predictability (Trevor-Roberts, 2006), because they enable employees to obtain more self-beneficial information and to enhance their self-perception through self-enhancement outcomes. Therefore, these behaviors constitute PFPB, satisfying the motivation to increase predictability. In addition, studies have found that PU can trigger self-interest motivation and corresponding behaviors (Feng et al., 2025). Given that PFPB is inherently self-beneficial and self-focused (Belschak and Den Hartog, 2010; Deci and Ryan, 2000), this finding supports our prediction that PU positively influences PFPB.

In general, we hypothesize that PU differentially affects proactive behaviors with different foci:

*H3a:* PU has a negative effect on POPB.

*H3b:* PU has a negative effect on PSPB.

*H3c:* PU has a positive effect on PFPB.

### 2.4 The moderating mediation effect

As mentioned, perception of uncertainty will reduce POPB and PSPB, but increase PFPB. Hence, we predict that media richness can moderate the indirect effect of telework intensity on proactive behavior via the perception of uncertainty. Thus, we propose moderated mediation hypothesis:

*H4a:* Media richness moderates the negative effect of telework intensity on POPB via PU, such that the indirect effect is weaker when media richness is high rather than low.

*H4b:* Media richness moderates the negative effect of telework intensity on PSPB via PU, such that the indirect effect is weaker when media richness is high rather than low.

*H4c:* Media richness moderates the positive effect of telework intensity on PFPB via PU, such that the indirect effect is weaker when media richness is high rather than low.

## 3 Method

### 3.1 Participants and procedures

We collected data from employees and their direct leaders across multiple provinces in China, including Shandong, Beijing, Shanghai, Jiangsu, Shanxi, and Jilin. To mitigate common method bias, we employed a two-wave, multi-source design. At Time 1, questionnaires were distributed to 439 employees measuring telework intensity, media richness, and demographics. We received 422 responses (96.13% response rate). Two weeks later at Time 2, the 422 employees were surveyed again to report their perception of uncertainty, yielding 343 responses (81.28%). Simultaneously, we distributed questionnaires to 86 leaders to evaluate their subordinates’ proactive behavior, receiving 69 completed leader surveys (80.23%). After matching employee and leader data using initials and excluding invalid responses with missing data or failed attention checks, we obtained a final sample of 212 matched leader-employee dyads (61.8% effective response rate).

Our sample consists of 212 employees matched with 81 supervisors. On average, each supervisor evaluated 2.62 subordinates. Of the 212 employee participants, 128 (60.38%) were male and 84 (39.62%) were female. Age was concentrated in the 26–35 range (121, 57.07%), followed by those aged 36–45 (62, 29.25%), 25 or younger (15, 7.08%), and 46 or older (14, 6.60%). Most participants held a bachelor’s degree (127, 59.90%), with the remainder holding an associate degree (64, 30.19%), a master’s or higher (16, 7.55%), or a high school diploma or less (5, 2.36%). Organizational tenure was distributed as follows: 1–3 years (59, 27.83%), 3–5 years (56, 26.41%), 5–10 years (42, 19.81%), 1 year or less (28, 13.21%), and more than 10 years (27, 12.74%).

### 3.2 Measures

To ensure scale generalizability, we adapted established measures from prior research. Scales originally in English were translated into Chinese following the back-translation procedure (Brislin, 1970). All items were rated on a 5-point Likert scale ranging from “strongly disagree” to “strongly agree.” All measurement items are provided in Appendix A.

#### 3.2.1 Telework intensity

Employees reported their telework intensity using the average weekly telework hours, following Golden and Veiga (2005).

#### 3.2.2 PU

Employees rated PU using the three-item scale from Camps et al. (2012). A sample item is “I feel uncertain about my present job”. The Cronbach’s alpha was 0.869.

#### 3.2.3 Proactive behavior

Leaders rated the employees’ POPB, PSPB and PFPB by using scales from Belschak and Den Hartog’s (2010) Employee Proactive Behavior Scale. For POPB, there are 3 items, and the Cronbach’s alpha was 0.797. A sample item is “At work, he/she will take the initiative to come up with ideas to solve the company’s problems”. For PSPB, there are 4 items, and the Cronbach’s alpha was 0.802. A sample item is “At work, he/she will take the initiative to share knowledge with colleges”. For PFPB, there are 4 items, and the Cronbach’s alpha was 0.823. A sample item is “At work, he/she will take the initiative to realize his/her personal goals at work”.

#### 3.2.4 Media richness

Employees rated the media richness using the adaptation of the media richness 4-item scale developed by Carlson and Zmud (1999). A sample item is “The communication media used during telework allow my team members and me to give and receive timely feedback”. The Cronbach’s alpha was 0.908.

#### 3.2.5 Control variables

Following prior research demonstrating that gender, age, education, and tenure influence POPB (Parker et al., 2010), we controlled for these demographic variables to rule out potential confounding effects.

## 4 Results

### 4.1 Measurement model test

Before testing our hypotheses, we used Mplus 8.3 to conduct Confirmatory factor analysis (CFA) to ensure that our focus variables were distinct. A five-factor basic model and four alternative models were developed. The result (see Table 1), indicated that the 5-factor model showed an appropriate fit to the data (*χ^2^/df* = 1.760, CFI = 0.953, TLI = 0.943, RMSEA = 0.063, SRMR = 0.041) and showed a significantly better model fit than all the alternative models. Given that the three proactive behavior dimensions were measured using the same scale, we further compared a three-factor model against a one-factor model to examine their distinctiveness. The three-factor model showed better fit (CFI = 0.949, TLI = 0.932, RMSEA = 0.086, SRMR = 0.039) than the one-factor model (CFI = 0.941, TLI = 0.927, RMSEA = 0.089, SRMR = 0.042). These findings provided support for construct distinction.

**Table 1:** Confirmatory factor analysis.

Model	Factor	χ^2^	df	CFI	TLI	RMSEA	SRMR
5-factor model	PU, POPB, PSPB, PFPB, MR	230.583	125	0.953	0.943	0.063	0.041
4-factor model 1	PU + POPB, PSPB, PFPB, MR	677.604	129	0.756	0.711	0.142	0.182
4-factor model 2	PU + PSPB, POPB, PFPB, MR	654.483	129	0.767	0.723	0.139	0.188
4-factor model 3	PU + PFPB, POPB, PSPB, MR	805.968	129	0.699	0.643	0.157	0.208
4-factor model 4	PU + MR, POPB, PSPB, PFPB	582.805	129	0.798	0.761	0.129	0.113
4-factor model 5	PU, POPB + PSPB, PFPB, MR	245.368	129	0.948	0.939	0.065	0.043
4-factor model 6	PU, POPB + PFPB, PSPB, MR	253.803	129	0.945	0.934	0.068	0.047
4-factor model 7	PU, POPB + MR, PSPB, PFPB	717.860	129	0.738	0.690	0.147	0.211
4-factor model 8	PU, POPB, PSPB + PFPB, MR	255.596	129	0.944	0.933	0.068	0.046
4-factor model 9	PU, POPB, PSPB + MR, PFPB	688.969	129	0.751	0.705	0.143	0.215
4-factor model 10	PU, POPB, PSPB, PFPB + MR	826.306	129	0.690	0.633	0.160	0.229
3-factor model 11	PU, POPB + PSPB + PFPB, MR	265.629	132	0.941	0.931	0.069	0.049

*N* = 212; PU = the perception of uncertainty; POPB = pro-organizational foci proactive behavior; PSPB = Prosocial foci proactive behavior; PFPB = Pro-self foci proactive behavior; MR = Media richness.

### 4.2 Descriptive statistics

Table 2 displays the results of means, standard deviations, and correlations of the variables in this study. As expected, telework intensity was positively correlated with PU (*r* = 0.205, *p* < 0.01). PU was both significantly correlated with POPB and PSPB (respectively *r* = −0.275, *p* < 0.01; *r* = −0.228, *p* < 0.01). These findings provided initial support for our hypotheses. However, the relationship between PU and PFPB was not significant. Thus, we carried out path analysis to examine all hypotheses using Mplus 8.3.

**Table 2:** Descriptive statistics and correlations.

Variable	*M*	*SD*	1	2	3	4	5	6	7	8	9
Gender	1.40	0.49	1								
Age	2.35	0.71	−0.037	1							
Education	2.73	0.63	−0.046	0.048	1						
Tenure	2.91	1.23	−0.059	0.530**	−0.032	1					
Telework intensity	13.39	11.52	−0.175*	0.131	−0.011	0.059	1				
PU	2.19	0.84	−0.052	0.068	−0.060	0.121	0.205**	1			
POPB	3.92	0.77	−0.054	−0.017	−0.004	−0.044	−0.201**	−0.275**	1		
PSPB	3.94	0.73	−0.058	−0.050	−0.037	−0.030	−0.133	−0.228**	0.747**	1	
PFPB	3.88	0.75	0.001	−0.003	−0.021	−0.021	−0.208**	−0.134	0.841**	0.765**	1
MR	4.25	0.74	0.001	−0.105	0.066	−0.078	0.109	−0.133	0.121	0.103	0.010

*N =* 212; PU = the perception of uncertainty; POPB = pro-organizational foci proactive behavior; PSPB = Prosocial foci proactive behavior; PFPB = Pro-self foci proactive behavior; MR = Media richness. ^*^p < 0.050. ^**^p < 0.010.

## 5 Hypothesis test

### 5.1 The moderated effect

To further test our hypotheses, we performed analyses via Mplus 8.3. We first examined the main and moderating effects for Hypotheses 1 and 2. All continuous variables were standardized before regression to reduce multicollinearity. We set PU as the dependent variable, telework intensity as the independent variable, media richness as the moderator, and gender, age, education and tenure as control variables. The interaction term of telework intensity and media richness was included to test the moderation. We also conducted a simple slope test with 5,000 bootstrap resamples to compare the focal relationship at high (+1 SD) and low (−1 SD) media richness levels. The analytical results are presented in Tables 3, 4.

**Table 3:** Results of path analysis.

Variables	The perception of uncertainty				The perception of uncertainty			
	*B*	SE	*t*	*p*	*B*	SE	*t*	*p*
Constant	2.040^**^	0.327	6.240	0.000	2.179^**^	0.311	7.003	0.000
Control variables								
Gender	−0.023	0.115	−0.199	0.842	0.021	0.112	0.186	0.853
Age	−0.022	0.091	−0.244	0.807	−0.085	0.089	−0.962	0.336
Education	−0.071	0.083	−0.852	0.394	−0.037	0.080	−0.456	0.648
Tenure	0.080	0.052	1.534	0.125	0.105^*^	0.052	2.000	0.045
Predictor								
Telework intensity	0.014^**^	0.005	2.975	0.003	0.194^**^	0.056	3.463	0.001
Media richness					−0.206^**^	0.087	−2.355	0.019
Telework × Media richness					−0.223^**^	0.090	−2.478	0.013
*R* ^2^	0.057				0.125^**^			

*N* = 212. ^*^p < 0.050. ^**^p < 0.010.

**Table 4:** Moderation effect (Based on 5,000 bootstrapping).

Paths	Estimate	95%CI
Telework intensity—the perception of uncertainty		
High media richness (+1*SD*)	−0.029	[−0.206, 0.151]
Low media richness (-1*SD*)	0.417	[0.227, 0.676]
Difference	−0.446	[−0.841, −0.152]

Specifically, Hypothesis 1 proposes that telework intensity can positively affect PU. Table 3 shows that the effect of telework intensity on PU was significant (*B* = 0.014, *p* < 0.01). Thus, Hypothesis 1 was supported. Hypotheses 2 proposes that media richness can moderate the relationship between telework intensity and PU. The results (see Table 3) based on 5,000 bootstrapping samples indicated that, after controlling the effects of control variables, the interaction term of telework intensity and media richness significantly influenced PU (*b* = −0.233, *p* < 0.050). Table 4 showed that telework intensity significantly affected PU when media richness was low (*b* = 0.417, 95% CI = [0.227, 0.676]). However, the effect was not significant when media richness was high, which means that the positive effect of telework intensity on PU only existed when media richness was low, but was nonexistent if media richness was high. In addition, the difference between the two effects was significant (*b* = −0.446, 95% CI = [−0.841, −0.152]). Thus, Hypothesis 2 was supported.

### 5.2 The moderated mediation effect

Next, we tested the differentiated effects of PU on the three foci proactive behaviors (Hypotheses 3a-3c) and the moderated mediation effects (Hypotheses 4a-4c) using a full path model in Mplus 8.3. We generated 5,000 bootstrap samples and calculated 95% bias-corrected confidence intervals (CIs). Effects were considered significant when the 95% CIs excluded zero. Demographic variables were controlled in all path models throughout the analysis. The analytical results are presented in Tables 5, 6.

**Table 5:** Results of path analysis.

Variables	Pro-organizational foci proactive behavior				Prosocial foci proactive behavior				Pro-self foci proactive behavior			
	*B*	SE	*t*	*p*	B	SE	*t*	*p*	B	SE	*t*	*p*
Constant	4.737^**^	0.374	12.679	0.000	4.811^**^	0.358	13.443	0.000	4.260^**^	0.392	10.872	0.000
Control variables												
Gender	−0.153	0.110	−1.385	0.166	−0.124	0.105	−1.174	0.240	−0.052	0.111	−0.472	0.637
Age	0.042	0.089	0.473	0.636	−0.041	0.083	−0.496	0.620	0.033	0.093	0.355	0.723
Education	−0.042	0.081	−0.515	0.606	−0.060	0.079	−0.762	0.446	−0.038	0.083	−0.463	0.643
Tenure	−0.012	0.048	−0.249	0.803	0.019	0.046	0.421	0.674	−0.005	0.047	−0.101	0.919
Predictor												
Telework intensity	−0.141^*^	0.057	−2.488	0.013	−0.075	0.051	−1.473	0.141	−0.146^*^	0.057	−2.555	0.011
Media richness	0.033	0.065	0.502	0.616	−0.008	0.070	−0.113	0.910	−0.037	0.073	−0.510	0.610
Telework intensity × Media richness	−0.132	0.078	−1.693	0.090	−0.166^*^	0.074	−2.253	0.024	−0.133	0.081	−1.639	0.101
Mediator												
The perception of uncertainty	−0.242^**^	0.081	−2.971	0.003	−0.213^**^	0.071	−3.012	0.003	−0.115	0.076	−1.516	0.129
*R* ^2^	0.144^**^				0.114^*^				0.079			

*N* = 212. ^*^p < 0.050. ^**^p < 0.010.

**Table 6:** Moderated-mediation Effect (Based on 5,000 bootstrapping).

Paths	Estimate	95%CI
Telework intensity—the perception of uncertainty—pro-organizational foci proactive behavior		
Mediation effect	−0.047	[−0.103, −0.011]
Moderated-mediation effect		
High media richness (+1 *SD*)	0.007	[−0.038, 0.060]
Low media richness (-1 *SD*)	−0.101	[−0.218, −0.026]
Difference	0.108	[0.025, 0.266]
Telework intensity—the perception of uncertainty—prosocial foci proactive behavior		
Mediation effect	−0.041	[−0.091, −0.010]
Moderated-mediation effect		
High media richness (+1 *SD*)	0.006	[−0.034, 0.052]
Low media richness (-1 *SD*)	−0.089	[−0.195, −0.025]
Difference	0.095	[0.024, 0.240]
Telework intensity—the perception of uncertainty—pro-self foci proactive behavior		
Mediation effect	−0.022	[−0.067, 0.004]
Moderated-mediation effect		
High media richness (+1*SD*)	0.003	[−0.016, 0.043]
Low media richness (-1*SD*)	−0.048	[−0.142, 0.010]
Difference	0.051	[−0.008, 0.178]

Specifically, the result (see Table 5) indicated that PU direct negatively affected POPB (*b* = −0.242, *p* < 0.010). Table 6 showed that the indirect effect of telework intensity on POPB was significantly negative when media richness was low (*b* = −0.101, 95% CI = [−0.218, −0.026]), but was nonexistence when media richness was high. The difference between two indirect effect was significant (*b* = 0.108, 95% CI = [0.025, 0.266]). Thus, Hypothesis 3a and 4a were supported.

For PSPB, PU direct negatively affected PSPB (*b* = −0.213, *p* < 0.010). In addition, Table 6 showed that the indirect effect of telework intensity on PSPB was significantly negative when media richness was low (*b* = −0.089, 95% CI = [−0.195, −0.025]), but nonexistent when it was high. The difference between two indirect effect was significant (*b* = 0.095, 95% CI = [0.024, 0.240]). Thus, Hypothesis 3b and 4b were supported.

However, the direct effect of PU on PFPB was not significant (see Table 5), and the moderated indirect effect on the relationship between telework intensity and PFPB was not significant yet. Thus, Hypothesis 3c and 4c were not supported.

## 6 Discussion

### 6.1 Discussion of findings

Taken together, our findings paint a clear picture: telework intensity triggers PU, which in turn dampens externally focused proactive behaviors while leaving self-directed development intact. Critically, however, these negative effects are not inevitable. When media richness is high, the telework-uncertainty link dissolves, and the indirect negative effects on proactive behaviors are fully attenuated. This suggests that the psychological cost of telework is driven not simply by telework hours, but also shaped by whether the communication environment can compensate for the loss of physical presence. Without rich media, telework breeds uncertainty and undermines proactive contributions; with rich media, these adverse effects are virtually eliminated.

Specifically, our findings reveal that telework intensity is positively associated with PU, suggesting that an important psychological mechanism through which telework intensity shapes employee behavior is the loss of informational and evaluative anchors. This is important because uncertainty directly shapes how employees interpret their work environment and decide whether to invest effort. Our results extend uncertainty management theory to include insufficient external information and unclear self-perceptions (Van den Bos, 2001), thereby complementing existing accounts of telework experiences (Adisa et al., 2023; Blind et al., 2024; Sardeshmukh et al., 2012; Tsai, 2025). Our results also provide quantitative support for Jämsen and Sivunen’s (2025) qualitative proposition that remote organizing exacerbates uncertainty.

In terms of behavioral outcomes, we find that telework intensity indirectly reduces POPB and PSPB but leaves PFPB stable. This pattern aligns with evidence that telework intensity impairs externally focused behaviors (e.g., OCB; Sun et al., 2024; Wang et al., 2025; Wörtler et al., 2021) but not self-directed development (Wang et al., 2025). Notably, the reason why PFPB remains unaffected may be that it stems from intrinsic motivations such as self-actualization and work commitment (Deci and Ryan, 2000) as well as personal career plans (Savickas, 2005), which are less susceptible to external disruptions. Additionally, this finding may also be partly explained by the limited observability of PFPB to others. In this study, we adopted a multi-source design in which perceived uncertainty and telework intensity were assessed by employees themselves, while all three foci proactive behaviors were rated by supervisors instead of employees to minimize common method bias. However, PFPB is inherently less visible to supervisors than POPB and PSPB, particularly in the telework context where direct observation is limited. One possible explanation for the nonsignificant PFPB result is therefore that the limited observability of PFPB may have reduced its empirical distinctiveness from POPB and PSPB in supervisor ratings. As detailed in the Limitations section, this measurement issue may have attenuated the detectability of the PU–PFPB relationship. Nevertheless, our results do support the empirical distinctiveness between POPB and PSPB, which is consistent with Belschak and Den Hartog's (2010) original validation. The findings regarding PFPB thus offer new insights into their framework in the telework context.

Finally, media richness moderates telework intensity’s positive effect on PU and its indirect negative effects on POPB and PSPB. This finding reveals that the psychological cost of telework depends not simply on how much employees work remotely, but on whether their communication environment compensates for the loss of organizational signals. When media richness is high, telework no longer generates uncertainty, and the downstream negative effects on proactive behaviors are eliminated. This pattern contrasts with research on technology-induced stress (Buzás et al., 2025; Dutta and Mishra, 2024; Lal et al., 2023) and supporting the buffering role of rich communication (Workman et al., 2003). Moreover, our examination of multi-feature digital communication moves beyond a narrow focus on single-channel richness (e.g., e-mail richness; Higa et al., 2000).

### 6.2 Theoretical implications

This study makes three theoretical contributions.

First, we extend telework outcome research from conventional indicators, such as performance, work attitudes, and well-being, to proactive behavior (e.g., Leroy et al., 2021; Nowrouzi-Kia et al., 2024; Sardeshmukh et al., 2012). More importantly, by demonstrating that telework-induced uncertainty impairs externally focused proactive behaviors, including those targeting the organization (POPB) and those targeting others (PSPB), we show that externally focused proactive behaviors are consistently constrained by uncertainty regardless of whether the beneficiary is the organization or others. This finding provides a more nuanced understanding of how telework intensity affects proactive behavior by distinguishing between pro-organizational and prosocial motives based on the behavioral target. It also demonstrates that a behavior’s sensitivity to telework depends critically on its focus. This insight contributes to a more parsimonious understanding of how telework intensity shapes proactive behavior and highlights two distinguishable foci of proactive behavior in the telework context. In doing so, this study extends Belschak and Den Hartog’s (2010) typology by identifying the outward-directed nature of proactive behavior as a key boundary condition.

Second, we introduce uncertainty management theory to telework research, offering a new perspective distinct from prior resource-based or relationship-based theories (e.g., Bartel et al., 2012; Blind et al., 2024; Huo et al., 2023; Kröll et al., 2021). Whereas existing telework research has primarily focused on how telework depletes resources or disrupts social connections, uncertainty management theory shifts attention to the cognitive and motivational consequences of insufficient information and unclear expectations. By demonstrating that uncertainty mediates the relationship between telework intensity and externally focused proactive behaviors, including those targeting the organization and those targeting others, we extend uncertainty management theory beyond its traditional domains and establish it as a viable framework for understanding employee behavior in remote work arrangements.

Third, we identify media richness as a boundary condition, extending media richness theory to contemporary telework. While recent research often emphasizes technology-induced technostress or insecurity in telework (Buzás et al., 2025; Dutta and Mishra, 2024; Lal et al., 2023), earlier work has examined the characteristics of communication technologies themselves, showing that media richness and face-to-face interaction are related to commitment in telework and virtual teams (Workman et al., 2003). Moreover, our examination of multi-feature digital communication implies that in contemporary telework, where multiple communication tools are used simultaneously, media richness should be assessed as a composite of features rather than a single dimension.

### 6.3 Practical implications

This study offers several actionable insights for organizations implementing telework. First, because telework intensity increases uncertainty, organizations should establish clear remote interaction protocols, including defining communication norms, such as timing, channels, and content, to enhance predictability and controllability. Furthermore, strengthening feedback mechanisms and fostering virtual communities can also reduce interpersonal isolation and reinforce a climate of support.

Second, given that perceived uncertainty reduces externally focused proactive behaviors (POPB and PSPB) while leaving self-directed development (PFPB) intact, managers should pay particular attention to employees’ organization- and colleague-directed initiatives during telework. Additionally, providing resilience and adaptability training will equip employees with skills to manage remote work challenges.

Finally, because media richness attenuates the telework-uncertainty link and eliminates the indirect negative effects on proactive behaviors, investing in high-quality digital collaboration tools, such as video conferencing and shared document platforms, is a strategic necessity. Organizations should also provide training to ensure employees use these tools effectively, as the benefits of media richness depend on actual usage fluency rather than tool availability alone. When media richness is high, telework no longer generates uncertainty, and employees maintain their proactive contributions; when it is low, even moderate telework intensity can undermine externally focused proactivity. Therefore, improving media richness should be prioritized alongside telework policy design.

### 6.4 Limitations and future research direction

This study has several limitations. First, while uncertainty was validated as a mediator based on uncertainty management theory, our measure of perceived uncertainty was general in nature (Camps et al., 2012). Future research could employ more focused scales to capture distinct dimensions such as task, communication, role, performance-evaluation, or career-development uncertainty, or examine other mechanisms such as psychological strain or motivational channels.

Second, although media richness was identified as a moderator, other factors may further clarify the boundary conditions of telework’s effects. These include theoretically relevant variables such as organizational support practices, leadership communication styles, and employee self-regulation, as well as contextual factors not captured in this study, such as supervisor demographics (e.g., age, gender, tenure) and organizational characteristics (e.g., industry, firm size). Future research could investigate these variables to provide a more complete understanding of telework’s contingent effects and enhance the generalizability of our findings across different work settings.

Third, although CFA model comparisons indicated that the hypothesized five-factor model fit slightly better than the alternative four-factor models, we acknowledge that more rigorous discriminant validity tests (e.g., HTMT and Fornell-Larcker criterion) were not fully satisfied, particularly those pertaining to PFPB. Specifically, the Fornell-Larcker criterion did not support robust discriminant validity between PFPB and the other two dimensions (POPB and PSPB), which may have attenuated the detectability of the relationship between PU and PFPB. The three proactive behavior dimensions were all rated by supervisors—POPB and PSPB are overt and relatively easy to observe, while PFPB is covert and harder to evaluate objectively, which may lead raters to conflate the three constructs and inflate their inter-factor correlations. In addition, the cross-sectional nature of our data, with perceived uncertainty and proactive behavior measured at the same time point, limits causal inference and may also contribute to inflated associations among constructs. Future research could address these concerns by adopting longitudinal designs with clearer temporal separation, as well as mixed self-report and supervisor-rating designs that assess covert dimensions (e.g., PFPB) via self-reports and overt dimensions via supervisor ratings.

Fourth, because some supervisors rated multiple employees, observations may not be fully independent at the supervisor level. Although the within-supervisor cluster sizes were small, we did not adjust for this clustering in the analyses. Future studies should consider using cluster-adjusted standard errors or multilevel modeling.

Fifth, we treated telework intensity as a linear variable. This approach was grounded in our theoretical logic that the telework state itself triggers PU. However, we acknowledge that this assumption may oversimplify the relationship. It remains possible that the effect is nonlinear, or that a certain threshold exists for the psychological mechanisms to fully manifest. Future research could explore these possibilities by testing curvilinear patterns or comparing subsamples of low- versus high-intensity teleworkers.

## Data Availability

The raw data supporting the conclusions of this article will be made available by the authors, without undue reservation.
