# Supplementary material for: The impact of telework intensity on employee proactive behavior: based on the uncertainty management theory
Source: Front Psychol. 2026 Oct 29;17:1854454. doi: 10.3389/fpsyg.2026.1854454 (PMC13754888; doi:10.3389/fpsyg.2026.1854454)
Supplement: Supplementary file 1 [file Supplementary_file_1.docx]

**Appendix A: Survey Scales**

***Telework Intensity (Golden & Veiga, 2005)***

The average number of hours per week they consistently spent away from their office working as a telecommuter.

***the Perception of Uncertainty (Camps, 2012)***

1. I feel uncertain about this situation.
2. I feel unsure about my current job.
3. I feel uncertain about the relation with my supervisor.

***Pro-organizational Foci Proactive Behavior (Belschak & Den, 2010)***

At work, he/she will take the initiative to……

1. ……suggest ideas for solutions for company problems.
2. ……acquire new knowledge that will help the company.
3. ……optimize the organization of work to further organizational goals.

***Prosocial Foci Proactive Behavior (Belschak & Den, 2010)***

At work, he/she will take the initiative to……

1. ……share knowledge with colleagues.
2. ……take over colleagues’ tasks when needed even though s/he is not obliged to.
3. ……help orient new colleagues.
4. ……help colleagues with developing or implementing new ideas.

***Pro-self Foci Proactive Behavior (Belschak & Den, 2010)***

At work, he/she will take the initiative to……

1. ……find new approaches to execute his/her tasks so that he/she can be more successful.
2. ……acquire new knowledge that will help his/her career.
3. ……realize his/her personal goals at work.
4. ……take on tasks that will further his/her career.

***Media Richness (Carlson & Zmud, 1999)***

1. The communication media used during telework (e.g., WeChat, phone, video conferencing software, collaboration software, etc.) allow my team members and me to give and receive timely feedback.
2. The communication media used during telework (e.g., WeChat, phone, video conferencing software, collaboration software, etc.) allow my team members and me to tailor our messages to our own personal requirements.
3. The communication media used during telework (e.g., WeChat, phone, video conferencing software, collaboration software, etc.) allow my team members and me to communicate a variety of different cues (such as emotional tone, attitude, or formality) in our messages.

The communication media used during telework (e.g., WeChat, phone, video conferencing software, collaboration software, etc.) allow my team members and me to use rich and varied language (such as natural language, numbers, dialects, etc.) in our messages.
